# Supplementary material for: The Physicochemical Characterization and In Vitro Digestibility of Maple Sugar Sand and Downgraded Maple Syrups
Source: Foods. 2023 Sep 22;12(19):3528. doi: 10.3390/foods12193528 (PMC10572138; doi:10.3390/foods12193528)
Supplement: Supplementary file 1 [file foods-12-03528-s001.zip › foods-2598345-supplementary.pdf]

Supplementary Table S1. Organic and inorganic composition of maple syrup (mean ± standard deviation and 95% confidence interval).

Authors	[1]	[2] reported by [3]	Dumont (1996) re- ported by [3]	Perkins (un- published) re- ported by [3]	van den Berg (un- published) re- ported by [3]	[4]	[5]	[6]	[7]
General									
Number of sam- ples	80	—	—	—	—	Vary for each analysis	8	57	6
Origin of samples	Canada (QC, ON), [illegible] (VT, MA,WI, NH, MI)	—	—	—	—	Canada (QC)	Canada (ON)		Canada (QC)
Brix (°)	67.0 ± 1.6 (62.2-74.0)	66.5	—	—	62.0–68.0	66.6 (66.2-67.3)	67.4 ± 0.8	—	—
Moisture (%)	31.7 ± 2.9 (26.5-39.4)	33.5	—	—	—	—	—	—	—
pH	6.7 ± 0.5 (5.6–7.9)	—	—	5.5–7.3	6.4	(5.5-8.0)	6.9 ± 0.3	7.32—8.15	7.3 ± 0.5
Carbohydrates									
Sucrose (%)	68.0 ± 4.0 (51.7-75.6)	(58.5–65.8)	42.3–74.0	—	59.4–73.8	64.18 (60.75-67.67)	—	61.25–63.81	64.4 ± 3.8
Glucose (%)	0.43 ± 1.11 (0.00-9.60)	(0–7.3)	0.0–7.2	—	0.0–1.6	0.11 (0.0-0.39)	0.73 ± 0.27	0.06–0.48	0.44 ± 0.60
Fructose (%)	0.30 ± 0.54 (0.00-4.00)	Trace	0.0–6.8	—	0.0–1.1	0.14 (0.0-067)	0.09 ± 0.1	0.03–0.40	0.33 ± 0.48
Minerals and Metals									
Potassium (ppm)	2026 ± 375 (1055–2990)	(1300–3900)	541–4031	1600–2590	963–3319	2404.2 (973.1-3960.3)	2341.2 ± 215.74	–	1670 ± 213
Calcium (ppm)	775 ± 279 (266–1707)	(400–2800)	183–1943	600–1250	278–2494	785.3 (113.2-1660.0)	883.4 ± 191.5	–	1691 ± 1191
Magnesium (ppm)	167 ± 72 (10–380)	(12–360)	11–575	0–198	25–543	202.2 (10.20.-379.8)	205.1 ± 108.3	–	191 ± 67
Manganese (ppm)	—	(2–220)	< 1–252	0–117	0.01–223	20.5 (0.3-60.0)	5.7 ± 4.3	–	13.5 ± 20.1

Sodium (ppm)	—	(0–6)	< 1–261	0–27	0.01–492	14.4<br>(0.0-90)	—	–	10.58 ± 1.66
Phosphorus (ppm)	—	(79–183)	< 2–235	20–113	0.01–91	—	5.5 ± 1.8	–	–
Iron (ppm)	—	(0–36)	0–18	0–18	0.01–61	4.4<br>(0.0-21.6)	—	–	–
Zinc (ppm)	—	(0–90)	2–43	0–96	0–130	4.4<br>(0.0-12.1)	6.0 ± 0.9	–	3.79 ± 0.45
Aluminum (ppm)	—	—	—	—	0.01–18	4.8<br>(0.0-28.8)	—	–	–
Boron (ppm)	—	—	—	—	0.01–3	—	—	–	–
Sulfur (ppm)	—	—	—	—	0.01–100	—	—	–	–
Copper (ppm)	—	(0–2)	0–8	0–6	—	1.9<br>(0.0-9.9)	—	–	–
Tin (ppm)	—	(0–33)	—	0–24	—	—	—	–	–
Lead (ppm)	—	(0–0.25)	0–0.49	0–0.35	—	—	—	–	–
Cadmium (ppm)	—	—	0–0.09	0–0.07	—	—	—	–	–
Organic Acids									
Fumaric (%)	0.004 ± 0.002<br>(0.001–0.012)	0.006	0.0–0.13	—	—	0.006<br>(0.001-0.018)	—	<0.01-0.01	0.003 ± 0.001
Malic (%)	0.47 ± 0.11<br>(0.06–0.66)	0.141	0.32–0.90	—	—	0.46<br>(0.17-0.77)	—	0.42—0.54	0.53 ± 0.15
Citric (%)	—	0.015	—	—	—	0.026<br>(0.011-0.046)	—	—	–
Succinic (%)	—	0.012	0.0–0.26	—	—	0.018<br>(0.004-0.039)	—	—	0.16 ± 0.16
Polyphenolic content									
Total Phenolic Content (μg/g)	—	—	—	—	—	977<br>(340-2124)	857.1 ± 218.2		62.33 ± 5.18

QC: Quebec; ON: Ontario; VT: Vermont; MA: Massachusetts; WI: Wisconsin; NH: New Hampshire; MI: Michigan.

Supplementary Table S2. Organic and inorganic composition ranges of maple sugar sands.

Authors	[8]	[9]
pH	6.30 - 7.20	-
Calcium (%)	0.61 - 10.91	0.05 - 11.50
Potassium (%)	0.146 - 0.380	0.02 - 1.51
Magnesium (%)	0.011 - 0.190	0 - 1.45
Manganese (%)	0.06 - 0.29	-
Phosphorus (%)	0.03 - 1.18	-
Iron (ppm)	38 - 1.250	-
Copper (ppm)	7 - 143	-
Boron (ppm)	3.4 - 23	-
Molybdenum (ppm)	0.17 - 2.46	-
Free Acid (%)	0.07 - 0.37	-
Total malic acid (%)	0.76 - 38.87	0.16 - 46.49
Acids other than malic (%)	0.08 - 2.62	0.01 - 11.71
Undetermined material (%)	6.94 - 34.16	-
Calcium malate (%)	1.30 - 49.41	-
Sugars in dried samples (%)	33.90 - 85.74	4.34 - 85.22
Sugar sand in dried samples (%)	14.26 - 66.09	-

References

1. Stuckel, J.G.; Low, N.H. The chemical composition of 80 pure maple syrup samples produced in North America. *Food Res. Int.* **1996**, *29*, 373–379.
2. Morselli, M.F. Chemical composition of maple syrup. *National Maple Syrup Digest.* **1975**, *14*, 12.
3. Heiligmann, R.B.; Koelling, M.R.; Perkins, T.D.; Ohio State, U. *North American Maple Syrup Producers Manual*; Ohio State University: Ohio, OH, USA, **2006**.
4. PPAQ, *Industry Sheet Maple Syrup of Québec.* **2018**.
5. Singh, A.S.; Jones, A.M.; Saxena, P.K. Variation and correlation of properties in different grades of maple syrup. P*lant Foods Hum Nutr* **2014**, *69*, 50–56. https://doi.org/10.1007/s11130-013-0401-x.
6. Filteau, M.; Lagace, L.; Lapointe, G.; Roy, D. Maple sap predominant microbial contaminants are correlated with the physicochemical and sensorial properties of maple syrup. I*nt. J. Food Microbiol.* **2012**, *154*, 30–36. https://doi.org/10.1016/j.ijfoodmicro.2011.12.007.
7. Lagacé, L.; Camara, M.; Martin, N.; Ali, F.; Houde, J.; Corriveau, S.; Sadiki, M. Effect of the new high vacuum technology on the chemical composition of maple sap and syrup. *Heliyon.* **2019**, 5(6), 35.
8. Davis, D. R.; Gallander, J. F.; Hacskaylo, J.; Gould, W. A. (1963). The chemical composition of maple sugar sand. *Journal of Food Science.* **1963**, 28, 182–190.
9. Gallander, J.F.; Hacskaylo, J.; Gould, W.A.; Willits, C.O. *Environmental and Chemical Factors Associated with Maple Sugar Sand Formation;* Ohio Agricultural Research and Development Center: Wooster, OH, USA, **1967**.
